# Adolescents’ physical activity at recess and actions to promote a physically active school day in four Finnish schools

**DOI:** 10.1093/her/cyu030

**Published:** 2014-06-06

**Authors:** H. L. Haapala, M. H. Hirvensalo, K. Laine, L. Laakso, H. Hakonen, T. Lintunen, T. H. Tammelin

**Affiliations:** ^1^LIKES—Research Center for Sport and Health Sciences, 40720 and ^2^Department of Sport Sciences, University of Jyväskylä, 40014 Jyväskylä, Finland

## Abstract

The national Finnish Schools on the Move programme support schools with their individual plans to promote school-based physical activity (PA). We examined the changes in adolescents’ recess and overall PA in four lower secondary schools and described the school actions to promote students’ PA and the local contact persons’ perceptions of the effects. Recess and overall PA were assessed four times by anonymous questionnaires from students in grades 7–9 (*n* = 789) in 2010–12, and local contact persons (*n* = 7) provided information on school actions with diaries, interviews and surveys. Student data were analysed with descriptive statistics and chi-square tests, and school actions data were analysed with quantitative content analysis. The proportion of students who participated in physical activities at recess at least sometimes increased from 30% to 49% in physically active play and from 33% to 42% in ball games, mostly due to improvements in males’ participation. Females’ participation in recess activities increased in two schools with gender-specific physical activities or facilities. Overall PA levels declined slightly. Organized recess activities, student recess activators and equipment provision and sports facilities development were considered to have affected students’ PA positively. Solutions for getting females more physically active in the school setting are needed.

## Introduction

Physical activity (PA) has multiple positive benefits for the physical and mental health of adolescents [1, 2], as well as their cognition and academic performance [3, 4]. However, adolescents seem to insufficiently engage in PA; only one-third of males and one-fifth of females aged 13 years in Finland meet the recommendation of at least 60 min of moderate-to-vigorous intensity PA (MVPA) daily [5, 6]. Young people’s PA levels are likely to decline with increasing age [7, 8], and in Finland this decline during adolescence is particularly steep [9]. This raises concerns over public health, since PA levels track from childhood and adolescence into adulthood [10].

Since the majority of adolescents spend a considerable amount of their time in schools, this environment is an important setting for the promotion of health-related behaviours, such as PA [11–13]. In addition to physical education, recess is one of the main opportunities for students to be physically active within the school setting [14, 15]. Recess can be defined as ‘non-curriculum time allocated by schools between lessons for children to engage in leisure activities’ [16, p. 361], and like in this study, recess can also include lunch breaks during the school day. Despite the declines identified in PA and increases in sedentary time during recess [17], recess time can contribute up to 40% of students’ daily MVPA [16, 17], meeting the daily PA recommendations [16] and the school day MVPA [18].

According to the social–ecological model, factors in multiple levels have an influence on health-related behaviours, such as PA [19]. Therefore, the development and implementation of interventions should consider individual, social, environmental and policy domains. Adolescents themselves have emphasized social and physical environmental factors as key strategies to promote their PA participation, such as improving social support from peers, availability of activities and provision of organized activities at school [20].

One suggestion for the successful implementation of PA promotion in the school environment has been the whole-school approach [21]. All segments of the school day are considered for PA opportunities, and both physical and human resources are incorporated into the promotion practices [21]. However, school staff members’ perceptions of their role in promoting students’ PA [22] or their readiness for change [23] may vary between schools. Developed interventions could be more flexible and adjustable for implementation in schools, and this could help the school staff to adopt and sustain the promotion practices. Yet, there is limited information about school discretion and provision of individual solutions and how this could contribute to the success of the promotion.

The aims of this study were to (i) examine the changes in adolescents’ recess and overall PA in four lower secondary schools (grades 7–9), (ii) describe the promotion actions conducted in these schools which participated in the Finnish Schools on the Move programme and (iii) investigate the local contact persons’ perceptions of these actions’ effect on students’ PA.

## Method

### Background programme description

The data for this study were collected from four schools in the national, on-going Finnish Schools on the Move programme. This action programme aims to establish a physically active culture in Finnish comprehensive schools [24]. The unique feature of the programme is its bottom-up approach; schools and municipalities participating in the programme implement their own individual plans to increase PA during the school day. The schools are not required to implement any particular actions by the programme. During the course of the programme, schools were provided with further ideas on how to activate the school day in the national programme seminars. Schools were also given the opportunity to co-operate with an experienced mentor to support and help them in the promotion process.

The Finnish Schools on the Move programme is funded by the Ministry of Education and Culture and is organized by the Board of Education, regional state administrative agencies and various other organizations, and it is part of the Government Programme. During its pilot phase (2010–12), 21 local regional projects with a total of 45 schools and 10 000 students in grades 1–9 throughout Finland were involved [24]. The schools applied voluntarily with a project plan. The Ministry selected the participating schools based on the plan quality and to represent Finnish schools with versatility in geographical location, size, type of school (primary versus lower secondary) and national languages (Finnish and Swedish). In this study, we concentrate on four lower secondary school cases.

### Study population

Four lower secondary schools with high response rates, variety in school size and geographical location and promising action plans were selected for this study. The data were collected from students in grades 7–8 in 2010–11 and in grades 8–9 in 2011–12 (Table I). The students (mean age 14.1 ± 0.6 years at baseline) participated voluntarily, and questionnaires were conducted four times in the same classes without individual identification information (Table I). At the baseline measurement there were 789 (males: 46%), in the second measurement 789 (males: 47%), in the third measurement 791 (males: 45%) and in the fourth measurement 704 respondents (males: 47%). Due to the follow-up without individual identification, the study population could have partly changed during the course of the measurements. According to school information, the yearly student turnover was less than 3%. During their regular school day, students completed an anonymous self-report questionnaire with measures of study variables and demographic items. Researchers administered the questionnaire completion, monitored understanding and answered participants’ questions. The average response rates varied between 82% and 87% in schools A, B, C and D (Table I).

**Table I.** Basic characteristics of the schools A, B, C and D and the number of respondents from grades 7–9 and response rates (%) at measurement points 1–4

			Measurement points			
			1	2	3	4
School	School size, spring 2012 (*n* for students)	Urbanity	11/2010–2/2011, *n* (%)	4–5/2011, *n* (%)	11–12/2011, *n* (%)	4–5/2012, *n* (%)
A	619 (grades 1–9)	Semi-urban	85 (75)	98 (92)	89 (80)	91 (82)
B	301 (grades 1–9)	Rural	84 (90)	83 (87)	84 (88)	77 (84)
C	596 (grades 7–9)	Semi-urban	332 (88)	330 (90)	323 (91)	258 (73)
D	484 (grades 7–9)	Urban	288 (84)	278 (83)	295 (85)	278 (80)

### Ethics statement

The study protocol and consent procedure were approved by the Ethics Committee of the University of Jyväskylä, and all measurements were carried out in accordance with the Declaration of Helsinki. At the start of the study, the participants were informed of the study protocol and the purpose of the study in two ways: verbally and in writing on the participant information sheet on the cover page of the questionnaire. Participation was voluntary and no individual identification information was collected. The participants gave their assent/consent to participate in the study by returning the questionnaire to the researchers. Parental consent was not obtained. According to the Ethical principles of the Finnish National Advisory Board on Research Ethics, it is not necessary to request a guardian's permission in Finland if the head teacher of a school has determined that the study would produce useful information for the school and the study can be carried out as part of the normal activities of the school. Broad questionnaires that do not directly collect identifying information for research purposes can be carried out without the consent of parents [25].

### Measures

#### Physical activity (students)

‘Spending most recesses outdoors or indoors’ was measured with the question ‘where do you usually spend your school recess?’ The responsive alternatives were ‘outdoors’ or ‘indoors’.

‘PA at school recess’ was measured with a subscale of activities at recess. The question ‘what do you usually do at school recess?’ was followed by statements, of which the following two were used in this study: ‘I take part in physically active play’ and ‘I play ball games, for example football’. Students responded on a 4-point scale (1 = at all recesses, 2 = at most recesses, 3 = sometimes and 4 = never). The response alternatives ‘sometimes’, ‘at most recesses’ and ‘at all recesses’ were combined.

In order to evaluate the validity of recess PA measures, a separate smaller subsample in a different dataset from the Finnish Schools on the Move programme was used to compare these self-reported measures with objectively measured school-time PA. Altogether 229 students from grades 4–5 and 7–8 (42% boys) completed identical questionnaires to this study and wore ActiGraph GT1M or GT3X accelerometers for 7 consecutive days in autumn 2010. Objectively measured school-time MVPA (minutes/hour) had strong positive associations with physically active play and ball games (Table II).

**Table II.** Pearson’s correlation coefficients for self-reported physical activities at recess and objectively measured school-time MVPA (minutes/hour) in grades 4–5 and 7–8 students

	All (*n* = 229)	Boys (*n* = 95)	Girls (*n* = 134)
Physically active play	0.52**	0.61**	0.44**
Ball games	0.58**	0.63**	0.51**

**Correlation is significant at the 0.01 level.

‘Overall PA’ was measured with a question used earlier in WHO’s Health Behaviour in School-aged Children survey, and it has had high reliability [26, 27] and acceptable validity [28, 29]. The question measured MVPA in the previous week with at least 60 min of MVPA per day, and students responded on an 8-point scale ranging from 0 to 7 days.

#### School actions for a more physically active school day (local contact persons)

The data on school actions provided background information to interpret the changes observed in students’ PA participation. The local contact persons in the projects kept follow-up diaries of the operations at school, including the themes: basic information of the project, recess physical activities, trips, procurements and construction, theme days, active commuting to school, schooling and meetings and other actions implemented. The diaries were gathered four times during the follow-up period. Two researchers conducted recorded, theme-based, telephone interviews twice (1/2011, 5/2012) and Internet surveys twice (5/2011, 1/2012) with seven local contact persons in the projects. The questions of the interviews and surveys are presented in Table III. The questions for theme-based telephone interviews and Internet surveys and the themes for the follow-up diaries were compiled by three researchers from the research centre. Supplementary information on the changes in the structure of the school day during the programme was acquired by telephone from the local contact persons in 11/2013.

**Table III.** The questions of the telephone interviews and Internet surveys conducted with the local contact persons

	Telephone interview	Internet survey	Internet survey	Telephone interview
	1/2011	5/2011	1/2012	5/2012
Estimate how many percent of the students have participated in the project actions (in the primary school level and in the lower secondary school level).	X	X	X	
How have the students participated in the project actions? In which ways?	X			
What have been the biggest successes so far?	X	X		
What have been the biggest problems so far?	X	X		
What were the factors that pushed the project forward?		X	X	
What were the factors that hindered the project to move forward?		X	X	
What are your opinions on the effects of the project so far?	X	X		X
- From the students’/teachers’ and staff’s/school community’s and culture’s/network’s point of view?				X
What is your opinion on the co-operation you have had in your project with the school principal/teachers and other staff members/parents/municipality/voluntary sector (sports clubs, other organizations)?	X			
Which networks have been involved in the project, and how have they been committed to the project? (a list of possible networks provided).		X		
Estimate the role of the aforementioned networks to the project in practice so far (not involved/support in the background/active partner).		X		
How have you distributed the work of the project in your municipality?	X			
Have you co-operated with the mentor assigned for your project? If you have, in which ways?	X	X		
Have the teachers in your school attended the updating training provided by the University of Jyväskylä?				X
What kind of support would you like to have from the Finnish Schools on the Move programme organization?	X	X		
Other comments?	X	X	X	X

Notes: In addition to the interviews and surveys, the local contact persons reported the basic school actions in the follow-up diaries (gathered four times during the 2-year follow-up).

#### The structure of school days and recess periods

In the Finnish school system, the length of recesses or lunch break is not regulated by law, and the teaching time should be divided into appropriate teaching periods. Thus, in practice students in Finland are provided with several recesses daily, and schools get to arrange lessons and break times relatively independently.

The recess characteristics in our study schools are presented in Table IV. The schools had five to six recesses per school day, with a mean length of 14–16 min per recess. Lunch breaks were included in the total number of recesses, and their length varied from 30–40 min. The total recess minutes per school day were 95 min in schools A and D, and 85 min in schools B and C. In school A, all recesses changed to compulsory outdoor recesses in the second academic year 2011–12 in the programme. The school building in school B was under renovation between 2010 and 2013, and students were not allowed to stay indoors during recess. However, students were allowed to walk 200 m to a nearby sports hall and outdoor field for recess activities. In 2011–12, school B introduced a daily outdoor activity recess. School C had two compulsory outdoor recesses per school day in the first programme year, and one outdoor recess in the second programme year. In school D, there was no obligation to go outside the school building during recesses.

**Table IV.** Recess periods and total recess minutes during the school day before the programme and in the first and second programme year

	School A			School B^a^			School C			School D			
Recess number	Before	1st y	2nd y	Before	1st y	2nd y	Before	1st y	2nd y	Before	1st y	2nd y
1	5	5	5			5	12	12	12	10	10	10
2	15	15	15	15	15	5	8	8	8	10	10	10
3	**30**	**30**	**30**	**30**	**30**	**35**	**30**	**30**	**30**	**40**	**40**	**40**
4	15	15	15	15	15	10	15	15	15	15	15	15
5	15	15	15	15	15	25	15	15	15	10	10	10
6	15	15	15	10	10	5	5	5	5	10	10	10
Total min/school day	95	95	95	85	85	85	85	85	85	95	95	95

^a^In school B, the school building was under renovation between 2010 and 2013 and students were not allowed to stay indoors during recess. However, students were allowed to walk 200 m to a nearby sports hall and outdoor field for recess activities. 1st y = first programme year 2010–11. 2nd y = second programme year 2011–12. School lunch breaks presented in bold. Compulsory outdoor recesses are underlined.

### Analyses

The data on students’ PA measures obtained from the student surveys were distributed into males and females by schools and all schools together. The differences between genders were analysed separately with cross-tabulation and chi-square test for each PA measure (spending recess outdoors, physically active play at recess at least sometimes, ball at recess at least sometimes and overall PA with at least 5 active days per week with more than 60 min of MVPA per day). The statistical analyses were made with IBM SPSS Statistics version 20.

The goal of the analysis of school actions data was to describe the practices implemented to enhance students’ PA in the school setting. First, recorded telephone-interview data were transcribed by two researchers. The written data (96 pages of follow-up diaries, 34 pages of interviews and 6 pages of surveys) were then analysed by one researcher using quantitative content analysis. The data were classified into 26 different actions, and they were regrouped under six categories. These categories are presented in the form of a table (Table V). The local contact persons’ perceptions of the actions’ effects on students’ PA were also identified and marked in the subcategories.

**Table V.** Actions conducted in schools A, B, C and D to create a more physically active school day

Actions	School			
	A	B	C	D
Physical activities organized in the school setting				
Daily compulsory outdoor recess			X	
Longer recess period for PA		X	X^a^	
Gender-specific physical activities or facilities	X		X^a^	
Organized physical activities during recess	X^a^	X^a^	X	X
Afterschool activities/clubs	X	X	X	
Physically active morning openings	X			
Physical break activities in lessons			X	X
Physically active homework	X			
Whole-school events related to sports and PA	X	X^a^		X
Equipment and facilities for PA				
Outdoor equipment available	X^a^	X	X^a^	X
Indoor equipment available	X	X	X^a^	X
Sports facilities developed	X	X	X^a^	X^a^
Student involvement				
Students participate in the school PA working group	X			X
Students as recess activators/peer instructors at recess	X^a^	X^a^	X	X
Schooling the students on PA and instructing	X	X	X	X
Leadership and school staff engagement related to the project				
Principal’s support	X	X	X	X
Hired project worker	X			
Schooling the staff on PA	X	X^a^	X	X
More than only a few teachers involved in the project	X		X	
Staff working group for physical activity promotion	X		X	
Informing and co-operation related to the project				
Informing within the school	X	X	X	X
Co-operation with parents	X		X	
Co-operation with municipality and office-holders	X	X	X	
Co-operation with local sport clubs	X			
Experienced mentor supporting the project	X	X	X	
Other actions				
Physical activity monitoring for students	X	X		

^a^Actions that the local contact persons in each school perceived to have had a positive effect on students’ PA. PA, physical activity.

## Results

The local projects in this study consisted of one urban and one rural lower secondary school, and two schools were located in semi-urban areas of the country. The school sizes varied from 300 to over 600 students, and the response rates from grades 7 to 9 were on average between 82% and 87% (Table I). The number of respondents from both genders was rather equal in schools A, C and D, varying from 45% to 56% in each measurement. However in school B, the number of male respondents was fairly low, from 35% to 37% in all the measurements.

### Students’ PA

**Fig. 1.**
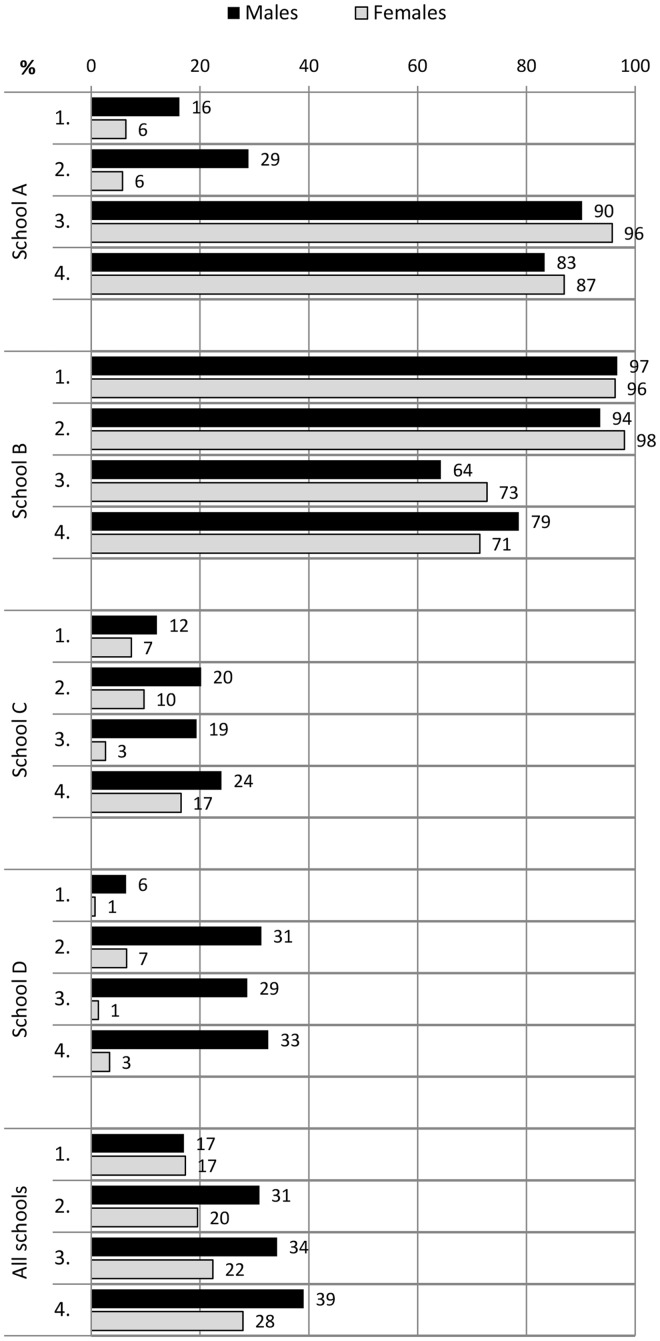
The proportion of students (%) who spent most recesses outdoors at the baseline and at the measurement points 2, 3 and 4 in schools A, B, C and D.

**Fig. 2.**
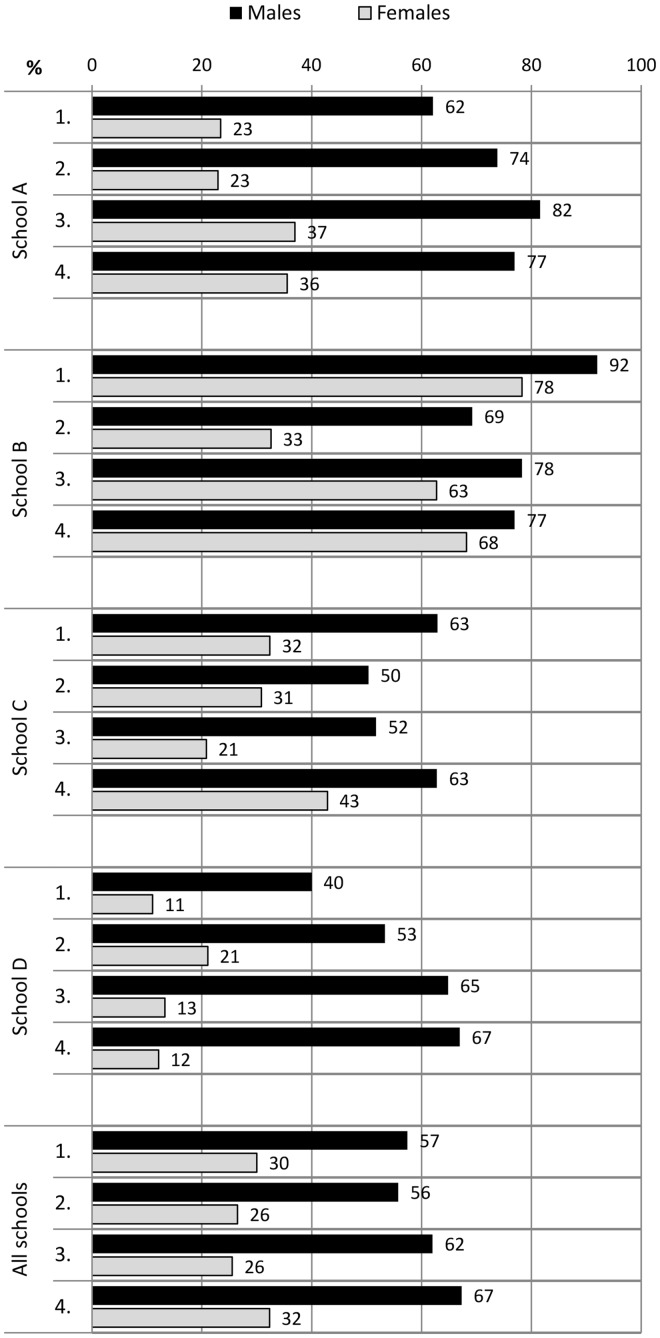
The proportion of students (%) who participated in physically active play at recess at least sometimes at the baseline and at the measurement points 2, 3 and 4 in schools A, B, C and D.

**Fig. 3.**
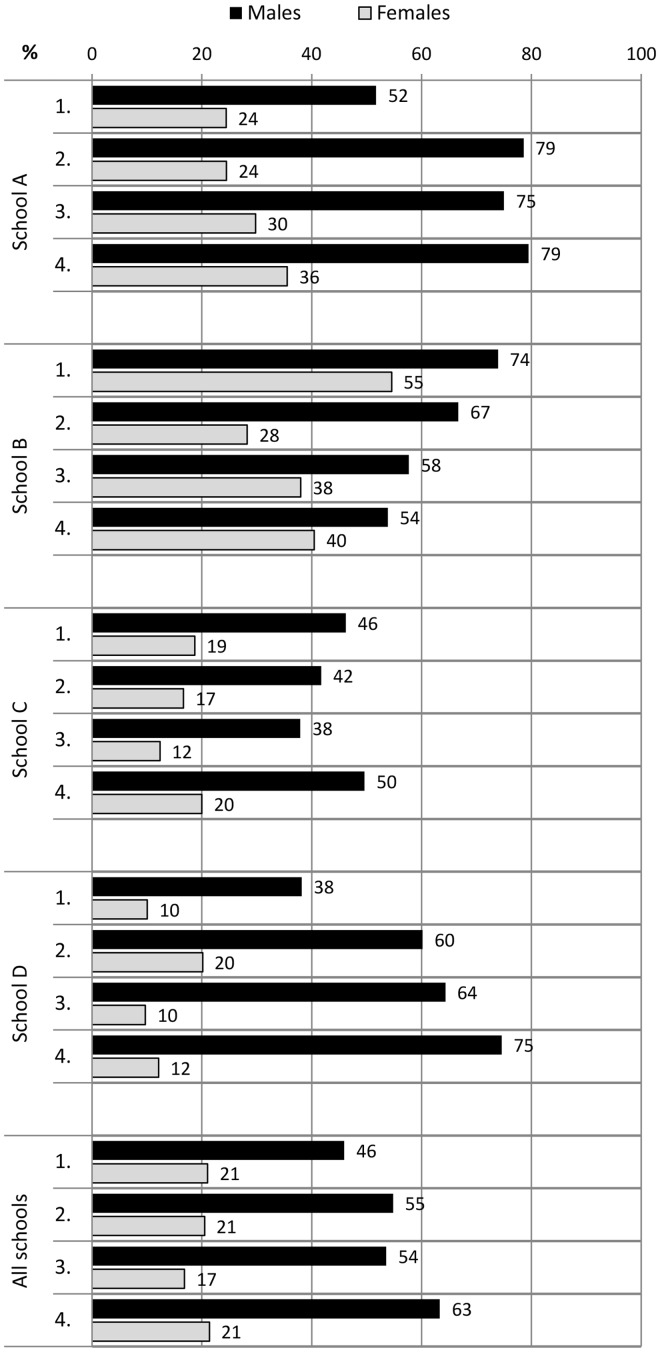
The proportion of students (%) who participated in ball games at recess at least sometimes at the baseline and at the measurement points 2, 3 and 4 in schools A, B, C and D.

**Fig. 4.**
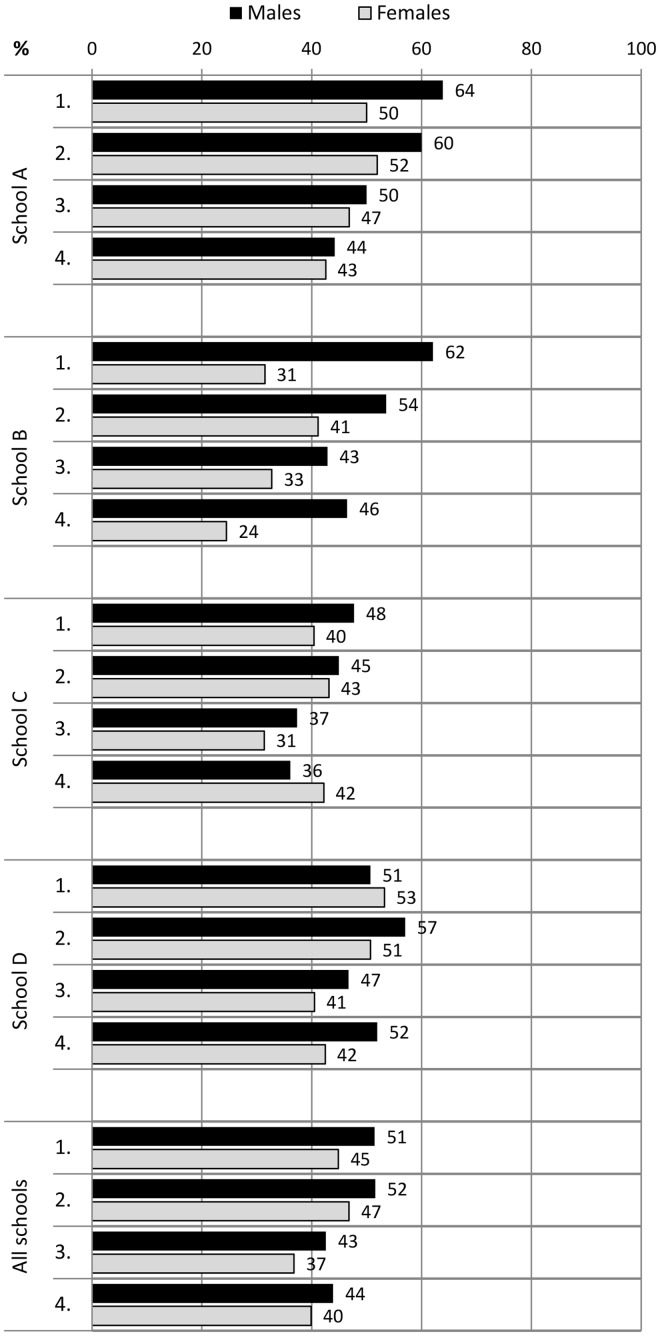
The proportion of students (%) with at least 5 active days a week (>60 min of MVPA per day) at the baseline and at the measurement points 2, 3 and 4 in schools A, B, C and D.

During the 2-year follow-up, the proportion of students who spent recess outdoors increased in all students from 17% to 33%. Males spent their recesses significantly more commonly outdoors than females in measurement points 2–4 (*P* < 0.003) (Fig. 1). The proportion of students who participated in physical activities at recess at least sometimes increased from 30% to 49% in physically active play and from 33% to 42% in ball games. These increases in both forms of PA at recess were mostly due to the improvements in males’ participation rates (Figs 2 and 3). Moreover, males participated more in recess activities than females in all measurement points (physically active play *P* < 0.001; ball games *P* < 0.001). Overall PA declined slightly in all students during the follow-up period (Fig. 4). The proportion of males who had at least 5 active days per week (with > 60 min of MVPA per day) decreased from 51% to 44%. The trend was similar in females (decline from 45% to 40%).

### School actions for a more physically active school day

The programme schools were able to independently plan the PA promotion considered to work in practice within the school community. The quantitative content analysis on the school actions data analysis condensed these actions into six main categories (Table V). ‘Activities within the school setting’ includes actions that transformed the structures of the school day and provided concrete opportunities for physical activities, such as longer recess periods and active morning openings. ‘Equipment and facilities’ describe the physical changes in the school environment, such as the provision of new game equipment, playground markings or playing fields both indoors and outdoors. ‘Student involvement’ refers to students’ opportunities to partake in the planning and decision-making of the physical activities implemented in the schools. ‘Leadership and staff engagement’ define the school personnel’s involvement in the process of making the school day more physically active. ‘Informing and co-operation’ describe the schools’ efforts to share knowledge and interest with different networks that have possibilities to affect schoolchildren’s physically active lifestyle, such as parents and local sports clubs. All the aforementioned categories included both practices implemented in the schools to directly promote students’ PA and factors behind the successful promotion process.

Most schools developed their equipment and facilities for PA. The majority of the physical activities carried out in the schools were non-curricular and outside lessons, such as recess and afterschool activities. Student involvement was applied especially when designing recess activities, and students were recruited in all the schools to activate other students at recess (recess activators/peer instructors). Organized recess activities both inside and outside the school building were also present in all schools. In addition, providing students with adequate and inspiring equipment for physical activities was one of the key strategies in the schools for PA promotion. The local contact persons considered organized recess activities, student recess activators and equipment provision and sports facilities development to have especially affected students’ PA positively (Table V).

Principals in all four schools supported the implementation of promotion actions, and all schools had their staff schooled about PA (Table V). Those schools that had a staff working group for PA promotion reported more than just a few teachers involved in the actions. Furthermore, information and communication within the school community were emphasized in all schools. Most of the schools also participated in mentoring, which meant contacting and developing ideas with an expert in the field of schoolchildren’s PA. The national programme had individually assigned a mentor for each local project, and the decision of its usage was left up to the projects themselves.

### School cases

In school A, there were organized recess activities and gender-specific physical activities and facilities available. This school also implemented physically active morning openings and physically active homework. A project worker was also hired to work on the PA promotion along with the staff. In this school, both males and females increased their participation in physical activities at recess. The proportion of students who spent their recesses outdoors increased in both genders, in males from 16% to 83% and in females from 6% to 87%. This was due to the implementation of compulsory outdoor recesses in the second programme year. The participation rates increased in physically active play from 62% to 77% in males and 23 to 36% in females, and in ball games from 52% to 79% in males and 24% to 36% in females. In overall PA, there was a decrease from 56% to 43% of students with at least 5 active days per week with both genders exhibiting a declining trend.

In school B, the local contact person was mostly responsible for the planning and implementation of new promotion actions. Longer recess periods with organized physical activities were introduced, and students were also instructed by their peers (recess activators). However, the high rates of 97% in males and 96% in females who spent most recesses outdoors declined to 79% and 71% during the follow-up, respectively. Moreover, the participation rates decreased in both physically active play and ball games among both genders (Figs 2 and 3). There was a decrease from 42% to 32% of students with at least 5 active days per week with both genders showing a declining trend.

In school C, longer recess periods and gender-specific PA opportunities during recess were implemented. Two daily outdoor recesses were introduced in the first programme year, and this changed to one outdoor recess in the second year. The school also had a number of staff members involved in a working group for PA promotion, and they developed networks with parents and the municipality office-holders. In this school, there was an increase in both genders’ participation in outdoor recesses (males from 12% to 24% and females from 7% to 17%). There was also an increase in participation among females in physically active play (from 32% to 43%) and among males in ball games (from 46% to 50%). The proportion of females with at least 5 active days per week increased slightly from 40% to 42%, whereas males in the most active category decreased from 48% to 36%.

In school D, organized physical activities at recess were introduced within the usual recess periods and physical break activities were implemented during lessons. Students also created a school PA working group in addition to schooling on PA and acting as recess activators. Nevertheless, the proportion of students who spent most recesses outdoors rose from 6% to 33% only in males. In addition, only males increased their participation rates in recess activities, in physically active play from 40% to 67% and in ball games from 38% to 75%. The proportion of males with at least 5 active days per week remained steady (from 51% to 52%) whereas the females’ proportion declined from 53% to 42%.

## Discussion

This study addressed the issues of changes in grade 7–9 students’ recess and overall PA during a 2-year follow-up period. We also explored which actions the schools implemented to promote school-based PA and which of these actions were considered to have had an effect on students’ PA by the local contact persons. In this study, students’ participation in physical activities at recess increased in three schools out of four during the follow-up both in physically active play and ball games. Spending recess outdoors also increased in these same schools, mostly due to increases in male participation. Organized recess activities, student recess activators and the provision of equipment and the development of sports facilities were considered to have had a positive effect on students’ PA.

Ridgers *et al.* [30] suggested that those adolescents who were involved in sport or PA during school recess had higher levels of light PA and MVPA in the long term. Accordingly, these increases in recess physical activities and outdoor recesses in this study are a promising trend. Time spent outdoors also correlate positively with PA levels in children [31], and outdoor facilities and equipment seem to be associated with adolescents’ break time activity at school [32]. The importance of equipment and facilities for students’ PA was also acknowledged by the local contact persons in our study. Increases in recess PA participation and outdoor activities also observed in our study might attenuate the decrease of PA levels during adolescence, and thereby, improve health outcomes.

The gender differences in recess activities were obvious in this study, supporting the previous findings of males’ physical activeness at recess [30, 33]. Male students in this study had significantly higher rates of participation in physical activities at recess at least sometimes compared with females, both in physically active play and in ball games. Male students also spent most of their recesses outdoors more often than females. In the recess and play context in primary schools, male students are shown to prefer rough and tumble play and aggression in play behaviours [34, 35]. Male students’ dominance for space in recess in a way most females avoid leaves less opportunities for females to be active [35]. Conflicts and teasing experienced by the opposite sex in play situations may contribute the lower participation rates in physical activities in adolescent females [36, 37]. However, the provision of non-competitive and innovative activities in the physical education setting has shown promising results in the promotion of adolescent females’ PA [38]. The importance of enjoyment and concerns of body attractiveness in adolescent females also support the provision of gender-specific activities [39]. Two schools (A and C) in this study were able to increase the participation of female students, and both schools applied separate physical activities at recess according to gender. The individualization and gender-sensitiveness in promotion actions could be one of the key strategies to promote PA in school among female adolescents [38].

The Finnish Schools on the Move programme has a unique approach to a more physically active school day. In this bottom-up approach, the schools and municipalities are given funding and the liberty to plan and implement actions they consider to be successful, unlike in most interventions. This approach reflects the overall Finnish Education Policy relying on customization, creativity, encouragement of risk-taking and shared responsibility and trust [40]. All the schools in the present study applied these possibilities by creating both educational and environmental/policy dimensions in promoting physical activities, as suggested by the social–ecological model [19]. Indeed, the implementation of multi-component approaches has shown promising results in improving adolescents’ PA levels [41], and a choice-based model with individualized school plans has shown positive results in the elementary level in both students’ PA levels and programme flexibility [42]. The opportunity to combine components freely within each school also allows the schools’ unique features to be taken into consideration, whether due to the readiness or knowledge of the staff, current facilities and equipment or already existing co-operation with different networks.

In this study, student involvement and empowerment to promote PA within the school environment was implemented in all four schools. Also, the local contact persons emphasized the role of student recess activators to have affected students’ PA positively. Adults in the school have the potential to either hinder or encourage PA for students [22], and for example reducing restrictions has been associated with increased school break time PA and decreased sedentary behaviour in adolescents [30]. Enabling students to be more proactive about their own PA in the school setting could especially motivate adolescents who in their developmental phase seek independence from adults and support from peers.

The strengths of this study include the focus on adolescents’ participation in physical activities at recess [43] and the description and explanation of the PA in the school setting with factors in the different systemic levels of the school. Since most follow-up studies and interventions concentrate on reporting the procedures and effects of the direct actions for school PA promotion, this study also widens the perspective to a whole-school approach. The content analysis revealed the involvement of school staff and students in the promotion actions, which is seldom reported. The bottom-up approach of this study has value, because it demonstrates how different approaches and solutions work for different schools.

The student follow-up with no identification information gave high response rates, but hindered possibilities for individual follow-up. This affects the ability to interpret the significance of the changes in PA measures. There were fewer male participants in school B, which might contribute to the results of the PA measures for this particular school. Self-report measures, especially of PA, can result in measurement errors and social desirability bias [44, 45]. However, the correlations between the self-reported recess physical activities with objectively measured school-time PA data were mostly strong (Table II). The nature of the analysis on school actions does not allow the extent and power of the actions presumed within each school. Given the half-structured nature and open questions in the interviews and surveys for the local contact persons, the school actions data might not be comprehensive and the classifications made might not be all-encompassing in school PA promotion. More than one researcher conducting the quantitative content analysis would have strengthened the analysis and provided more points of views.

In conclusion, we showed that students’ PA at recess and outdoor recesses increased the most in males. Further research is needed on the solutions for encouraging female adolescents to be more physically active. The lower secondary schools concentrated mostly on activating the recesses, and future challenges also lie in the activation of lessons and reducing sedentary behaviour within the school days.

## References

[1] Janssen I, Leblanc AG (2010). Systematic review of the health benefits of physical activity and fitness in school-aged children and youth. Int J Behav Nutr Phys Act.

[2] Rothon C, Edwards P, Bhui K (2010). Physical activity and depressive symptoms in adolescents: a prospective study. BMC Med.

[3] Hillman CH, Erickson KI, Kramer AF (2008). Be smart, exercise your heart: exercise effects on brain and cognition. Nat Rev Neurosci.

[4] Singh A, Uijtdewilligen L, Twisk JWR (2012). Physical activity and performance at school: a systematic review of the literature including a methodological quality assessment. Arch Pediatr Adolesc Med.

[5] Currie C, Zanotti C, Morgan A (2012). Social Determinants of Health and Well-Being Among Young People. Health Behaviour in School-Aged Children (HBSC) Study: International Report from the 2009/2010 Survey.

[6] Strong WB, Malina RM, Blimkie CJR (2005). Evidence based physical activity for school-age youth. J Pediatr.

[7] Riddoch C, Andersen LB, Wedderkopp N (2004). Physical activity levels and patterns of 9- and 15-yr-old European Children. Med Sci Sport Exerc.

[8] Currie C, Gabhainn SN, Godeau E (2008). Inequalities in Young People’s Health. HBSC International Report from the 2005/2006 Survey.

[9] Iannotti RJ, Kalman M, Inchley J, Currie C, Zanotti C, Morgan A (2012). Energy expenditure: moderate-to-vigorous physical activity. Social Determinants of Health and Well-Being Among Young People. Health Behaviour in School-Aged Children (HBSC) Study: International Report from the 2009/2010 Survey.

[10] Telama R (2009). Tracking of physical activity from childhood to adulthood: a review. Obes Facts.

[11] Dobbins M, Husson H, DeCorby K (2013). School-based physical activity programs for promoting physical activity and fitness in children and adolescents aged 6 to 18. Cochrane Database Syst Rev.

[12] Naylor P-J, McKay HA (2009). Prevention in the first place: schools a setting for action on physical inactivity. Br J Sports Med.

[13] Nettlefold L, McKay HA, Warburton DER (2011). The challenge of low physical activity during the school day: at recess, lunch and in physical education. Br J Sports Med.

[14] Beighle A, Morgan CF, Le Masurier G (2006). Children’s physical activity during recess and outside of school. J Sch Health.

[15] Jago R, Baranowski T (2004). Non-curricular approaches for increasing physical activity in youth: a review. Prev Med.

[16] Ridgers ND, Stratton G, Fairclough SJ (2006). Physical activity levels of children during school playtime. Sports Med.

[17] Ridgers ND, Timperio A, Crawford D (2012). Five-year changes in school recess and lunchtime and the contribution to children’s daily physical activity. Br J Sports Med.

[18] Ridgers ND, Saint-Maurice PF, Welk GJ (2011). Differences in physical activity during school recess. J Sch Health.

[19] Sallis JF, Cervero RB, Ascher W (2006). An ecological approach to creating active living communities. Annu Rev Public Health.

[20] Hohepa M, Schofield G, Kolt GS (2006). Physical activity: what do high school students think?. J Adolesc Health.

[22] Huberty J, Dinkel D, Coleman J (2012). The role of schools in children’s physical activity participation: staff perceptions. Health Educ Res.

[23] Ehlers DK, Huberty JL, Beseler CL (2013). Is school community readiness related to physical activity before and after the Ready for Recess intervention?. Health Educ Res.

[24] Tammelin T, Laine K, Turpeinen S Final Report on the Finnish Schools on the Move Programme’s Pilot Phase 2010–2012 [Report in Finnish, Summary in English].

[25] National Advisory Board on Research Ethics Ethical Principles of Research in the Humanities and Social and Behavioural Sciences and Proposals for Ethical Review.

[26] Roberts C, Tynjälä J, Komkov A, Currie C, Roberts C, Morgan A (2004). Physical activity. Young People’s Health in Context Health Behaviour School-Aged Children Study International Report from 2001/2002 Survey.

[27] Liu Y, Wang M, Tynjala J (2010). Test–retest reliability of selected items of Health Behaviour in School-aged Children (HBSC) survey questionnaire in Beijing, China. BMC Med Res Methodol.

[28] Booth ML, Okely AD, Chey T (2001). The reliability and validity of the physical activity questions in the WHO health behaviour in schoolchildren (HBSC) survey: a population study. Br J Sports Med.

[29] Ridgers ND, Timperio A, Crawford D (2012). Validity of a brief self-report instrument for assessing compliance with physical activity guidelines amongst adolescents. J Sci Med Sport.

[30] Ridgers ND, Timperio A, Crawford D (2013). What factors are associated with adolescents’ school break time physical activity and sedentary time?. PLoS One.

[31] Ferreira I, van der Horst K, Wendel-Vos W (2007). Environmental correlates of physical activity in youth—a review and update. Obes Rev.

[32] Haug E, Torsheim T, Sallis JF (2010). The characteristics of the outdoor school environment associated with physical activity. Health Educ Res.

[33] Ridgers ND, Salmon J, Parrish A-M (2012). Physical activity during school recess: a systematic review. Am J Prev Med.

[34] Pellegrini AD, Smith PK (1998). Physical activity play: the nature and function of a neglected aspect of playing. Child Dev.

[35] Blatchford P, Baines E, Pellegrini A (2003). The social context of school playground games: sex and ethnic differences, and changes over time after entry to junior school. Br J Dev Psychol.

[36] Slater A, Tiggemann M (2010). “Uncool to do sport”: a focus group study of adolescent girls’ reasons for withdrawing from physical activity. Psychol Sport Exerc.

[37] Slater A, Tiggemann M (2011). Gender differences in adolescent sport participation, teasing, self-objectification and body image concerns. J Adolesc.

[38] Camacho-Miñano MJ, LaVoi NM, Barr-Anderson DJ (2011). Interventions to promote physical activity among young and adolescent girls: a systematic review. Health Educ Res.

[39] Biddle S, Whitehead SH, O’Donovan TM (2005). Correlates of participation in physical activity for adolescent girls: a systematic review of recent literature. J Phys Act Health.

[40] Sahlberg P (2011). Finnish Lessons: What Can the World Learn from Educational Change in Finland.

[41] Van Sluijs EM, McMinn AM, Griffin SJ (2007). Effectiveness of interventions to promote physical activity in children and adolescents: systematic review of controlled trials. BMJ.

[42] Naylor P-J, Macdonald HM, Zebedee JA (2006). Lessons learned from Action Schools! BC—An “active school” model to promote physical activity in elementary schools. J Sci Med Sport.

[43] Parrish A-M, Okely AD, Stanley RM (2013). The effect of school recess interventions on physical activity: a systematic review. Sports Med.

[44] Sallis JF, Saelens BE (2000). Assessment of physical activity by self-report: status, limitations, and future directions. Res Q Exerc Sport.

[45] Shephard RJ (2003). Limits to the measurement of habitual physical activity by questionnaires. Br J Sports Med.

